# Gender and Socioeconomic Status as Factors of Individual Differences in Pre-University Students’ Decision-Making for Careers, with a Focus on Family Influence and Psychosocial Factors

**DOI:** 10.3390/ijerph18031344

**Published:** 2021-02-02

**Authors:** María del Carmen Olmos-Gómez, Mónica Luque-Suárez, Diego Becerril-Ruiz, Jesús Manuel Cuevas-Rincón

**Affiliations:** 1 Department of Research Methods and Diagnosis in Education, Faculty of Education and Sport Science, University of Granada, 52005 Melilla, Spain; jcuevas@ugr.es; 2 Department of Sociology, Faculty of Education and Sport Science, University of Granada, 52005 Melilla, Spain; 3 Department of Sociology, Faculty of Sociology, University of Granada, 18004 Granada, Spain; becerril@ugr.es

**Keywords:** family influence, secondary transition to higher education, career guidance, decision-making, psychosocial factors

## Abstract

The present study analyses the influence of family, specifically parents, on the career decisions of their children, and how gender and socioeconomic status influence this choice. Research was carried out using data obtained from a questionnaire administered to a representative sample of students who took the university entrance examination (PEvAU, its Spanish acronym) in Spanish North Africa. A quantitative research design was adopted in which multivariate analysis (MANOVA) was applied anda decision tree, which was also used for graphical and analytical analysis. The main results indicated a significant influence of parents on their children’s choices, this being the best predictor regarding the decision to take the said examination. It was also verified that their choice of Spanish Baccalaureate programme was significantly associated with their selected career path, and gender and socioeconomic status had a significant impact on that decision. These results lead to the need for further research on guidance from secondary education onwards. It would be interesting to analyse factors neglected thus far, such as those related to the cultural environment of students.

## 1. Introduction

Family is a central institution in the lives of all people. From birth or even before it, as a context for procreation, the family environment is inevitable for every individual. Despite the social transformations of the 20th century, families have learned how to adapt and survive, remaining fully involved in the multiple facets of individuals’ lives [1]. This, however, cannot be said of marriage, which was formerly closely connected to the concept of family, but nowadays does not have the characteristic of involving their descendants. Many couples have children without being married, either in a common-law relationship or sometimes facing the challenge of single parenting. Leaving this aside, the family context is inescapable.

A family exists prior to the individual, and it is within this context that the individual develops during the most crucial years of their education [2]. The influence, educational styles, and forms of families have a unique impact on the socialisation and development of a person [3]. In this sense, the general goal of this study is to understand the major role played by parents in the acceptance of problems and in decision-making processes [4]. Thus, it affirms the notion that the family unit is a key factor in children’s mental health [5].

Family is not only extended in time or present from the initial periods of socialisation, but it also creates a space of intimacy. The affective and emotional relationships that give substance to a family are a major key to understanding the influence and career guidance that it provides to individuals. Proof of the strength of family is that, despite multiple attempts, a more robust and universally valid alternative has not been found [6]. In this work, our objective is to understand the influence that family characteristics, especially those of parents, have on students’ career decisions. 

Some authors [7] established a close relationship between success in higher education and the student’s socioeconomic level of origin since families with higher socioeconomic status have more capacity to support and maintain the child’s career, providing information and resources for career planning and decision-making [8]. Bulgarelli, Rivera, and Fallas [9] define decision-making as the ability of a person to gather all personal information, the characteristics that each profession has, and the purpose of choosing one that provides personal and professional achievement. To this, we add the definition of a career as, in the words of Baglama and Uzunboylu [10], the “synthesis of work roles that a person experiences during their life”. Adolescents are indecisive, and thus, in the words of Elster [11], they assign subjective probabilities to their decisions given the lack of information about it, which in some cases makes parents lead their children to choose a career that does not fit their personality because of concern of the child choosing the wrong career, which causes adolescents to feel pressured by the expectations of parents. Economic dependence is added to this, which makes them comply with the choices of parents [12].

In those moments, students experience a period of indecision that is also detected by parents [13] and usually attributed to the immaturity of their children [14]. The influence that parents can have on their children in the educational field seems to be indirect [15,16,17]. Psychosocial factors that influence an adolescent’s decision-making are determined by other aspects that mutually influence each other, configuring the adolescent’s personality, such as self-concept, motivation, and self-esteem [18].

Whiston and Keller [19] specified two areas of family influence: one relates to their own characteristics, such as parents’ occupation or educational level, while the other focuses on a less tangible dimension that involves the family atmosphere or parents’ expectations for their offspring. Both processes are combined and interact to influence the educational decisions of children. During adolescence, the use of strategies and personal skills to cope with the challenges and demands of academic life is especially important, given that this is a critical period for the development of an adult personality [20]. It can also help prevent poor academic performance and improve their personal and academic development [21]. 

For parents and their interest in said decisions, it is important to provide support rather than taking direct action and transmitting these preferences to their children, trying to influence them in some way. There is, however, a palpable fear that rebellious adolescent feelings lead to a different choice just because it goes against their parents’ wishes [13]. Nevertheless, agreement between parents and children is a recurrent finding in research [22,23], with 7 out of 10 students making choices that correspond to what their parents desire for them [24]. In the opposite case, when children make choices different from those of their parents, acceptance and respect is the most common behaviour in the family context. This is a reasonable reaction since parents consider that their children are those responsible for making that choice, and thus the intention is not to impose or force their position on the adolescents [13,25,26].

One of the main variables related to parents is educational level. Children whose parents have a university degree are more likely to go to university. Approximately half of graduate parents have children with the same educational level [24]. These children study at university and mostly do so in the same field of study as that of one of their parents [13]. The aforementioned research clearly shows that, in the opposite case, nongraduate parents, there is also a general tendency for them wanting their children to study at university as a means of generational social mobility. 

Socioeconomic status is also a determining factor. The economic situation of a family impacts school attendance and dropouts, as well as access to university [27]. A difficult economic situation determines both the number of resources that can be invested in education and the need for children to work as soon as possible to provide income [28]. Therefore, it is understandable that a family’s economic circumstances are influential when considering applying to a university. 

Gender is another standard variable used in the analysis of educational career guidance. On the national level, men tend to opt for technological and experimental areas of knowledge, whereas women are more likely to choose social and legal sciences, health sciences, and humanities [13,29,30,31]. This difference is key to understanding their social and labour market integration. 

Gender differences are also observed in relation to preferred work values. Men choose economic well-being and being responsible for others, while women choose helping other people and work–life balance [32]. However, without considering other variables and gender diversity, internal values, vocation, or an interesting and dynamic job are the most powerful contextual influences [24,33,34].

The media, so present in today’s society, have unclear impact on student decision-making. Although some studies proved their influence on adolescents [35], they are hardly recognised as influential in others [24].

According to the latest Spanish research, friends seem to have a certain power of influence. Other research studies showed significant influence of the peer group [36,37,38], which has to do with its position as role model of which the behaviour and actions are closer to one’s own. Students recognise that friends are important, but not so much as influence in the decision, but rather as support once the choice has been individually made [31]. However, this study also noted that parents question the validity of statements about the indecisive influence of friends. 

Siblings are perceived as more influential in 6% of cases, with more significance for males (8%) than for females (5%) [24]. In that research, teachers (6%) show similar influence to that of siblings. If the role of teachers in the lives of students is considered, however, their influence is lower, a finding that was confirmed by other studies [31,39,40]. 

Another factor with potential influence is career guidance. This is regarded by students as important [41,42], yet they recognise that counsellors or advisors have generally had little impact on their decision-making [24,40,43]. 

Among all analysed factors, the little importance that is generally attributed to counsellors is surprising. Either students are very clear about what they want, and career guidance services do not lead to a substantial change in what they have already decided, or there is a lack of connection between counsellors and teenagers. Closely associated with this is, for instance, the limited use of e-guidance, that is, the necessary improvement and low adoption of information and communication technology (ICT) by counselling departments, academic institutions, and the educational administration [44].

In an increasingly globalised and multicultural world, the relevance of the cultural factor in the decision-making process is also important. Individualistic cultures are different from collectivist cultures, and that determines the family influence [45]. Culture is transmitted to children in the process of socialisation and orientates them towards different cultural parameters, even when living in the same society. These cultural factors are highly significant as a field of study, as they can affect the educational decisions of children.

On the basis of the above considerations, and as a novelty, this research paper aimed to determine whether there are differences in opinions among students regarding career decision-making according to gender (male or female) and socioeconomic status (high, middle, or low), using descriptive statistics and graphical and analytical results derived from decision tree analysis of questionnaire data, and the interaction between the studied groups through multivariate analysis of variance.

## 2. Materials and Methods 

### 2.1. Methods

In this study, a quantitative methodology was used since the aim was to determine, by means of a questionnaire, the perceptions of surveyed adolescents regarding correct decision-making at each stage of the educational process and its relationship with family and gender. The questionnaire was tested for its rigor, adaptability to context, validity and reliability, and for its capacity to offer objective data with respect to the case under study. Two essential psychometric properties of any measuring instrument—reliability and validity—were tested, and both showed excellent results. This research was based on a quantitative study of a descriptive nature, using a social analytical empirical research method [46].

### 2.2. Participants

A sample of 1302 students aged between 17 and 19 years in the second year of the Spanish Baccalaureate was used (46% male and 54% female). The examined programmes of Spanish Baccalaureate were science and technology (33.1%), humanities (30.6%), social sciences (31.7%), and art (4.6%). Participants represented 99% of the overall number of final-year students with baccalaureate education (27.5% had high socioeconomic status, 52.3% had middle socioeconomic status, and 20.2% had low socioeconomic status). These students came from seven secondary public schools located in Melilla, a Spanish North African city, where the principal social and economic enclave of the city is the service sector, especially the civil service, with no agricultural or industrial activity.

### 2.3. Instrument 

The members of a multidisciplinary team from the university’s career guidance centre (University of Granada) that specialises in the personal, professional, and academic counselling of pre-university and university students participated in this phase. They developed and validated a questionnaire to measure the way in which pre-university students’ decision-making for careers with a focus in family and psychosocial factor influences the university degree choices of Spanish Baccalaureate students. The questionnaire incorporated variables related to socioeconomic status and gender. The designed instrument followed the main theoretical foundations and international recommendations for the construction of tests. For data collection, the present study relied on the voluntary participation of students who took the university entrance examination (PEvAU) in the autonomous city of Melilla. Permission was granted to enter classrooms. 

For the questionnaire that was developed, the Social Survey 2010: Education and Housing in Andalusia (ESOC2010) [47], the Questionnaire of Areas of Basic Professional Academic Interests: CIBAP [48], and the Psychological and Learning Environments in Adaptive Contexts Questionnaire (QPLEC) [49] were considered. 

The first version of the questionnaire was designed according to the standards set by the American Association of Educational Research, the American Association of Psychology, and the National Council for Educational Evaluation in 2014. Afterwards, the content validity and consistency of the instrument were examined by expert judgement using the Delphi method [50] through 3 rounds of analysis by 8 experts in educational research. After these analyses, the adjustments and revisions suggested by the group of experts regarding the coherence, adequacy, and relevance of the items were given due consideration. The percentage of agreement among the experts in the first round was between 69% and 72%; in the second round, between 72% and 84%; and in the third round, over 89%. Items below 70% were removed. The final version was reduced to 22 items without including identification [51]. 

This later version of the questionnaire, consisting of 22 items, was administered to a pilot sample of students (*n* = 215) on similar study characteristics who were asked to indicate the correct answer. Of these items, 5 were sociodemographic in nature. This version was used to perform psychometric reliability and validity analysis. 

Reliability was calculated using Cronbach’s alpha with an alpha value of =0.912, which is an excellent value for ordinal data [52].

In order to test data validity, exploratory and confirmatory analysis was conducted.

Construct validity was established with exploratory factor analysis that allowed for us to order the data and facilitate the interpretation of correlations. The Kaiser–Meyer–Olkin (KMO) index was calculated [53] (KMO = 0.822), obtaining a value that was more than optimal. In addition, Bartlett’s test of sphericity was performed, showing a value that was significant at the 0.000 level, which corroborated that the conditions were appropriate to perform factor analysis. Next, we analysed variance between all analysed variables in the table of total variance, with a result of 3 factors that explained 59.078% of the variance. Lastly, criterion validity was established with confirmatory factor analysis that was conducted through multivariate regression using structural equation modelling (SEM). With this inferential analysis, we could relate response patterns to a set of latent factors that could not be directly observed (observed variables) but existed, and dimensions that represented them as variables of interest (factors) through goodness-of-fit criteria and indices based on the analysis of covariance structures of observed variables and those reproduced by the model [49], of which the values are detailed in Table 1. The comparative fit index (CFI) was 0.87, and the root mean square error of approximation (RMSEA) was 0.045, with a range of 0.044–0.076 and a confidence of 90%, forming an appropriate range [54].

### 2.4. Procedure

Before conducting the research, we gave necessary notice and obtained permission from the career guidance services and management teams of 7 Spanish North African secondary schools offering baccalaureate-level education. All educational institutions were sent an email informing them of the voluntary and anonymous nature of the study, along with its purposes and objectives. The ethical principles of the Declaration of Helsinki were followed throughout the study.

Subsequently, the questionnaire was administered to the students in both paper-based format and online using Google Forms, including a brief introduction by the researchers; then, the questions were read as a group to allow for clarification questions. The questionnaire was administered over July and September 2020. Questionnaires were distributed to the students and took approximately 15 min to be completed.

The information collection instrument contained 27 items grouped into 4 sections. The first section gathered participants’ personal data through 5 items: gender (male and female), age, socioeconomic status (a basic monthly salary of EUR 1000, a medium monthly salary of EUR 1000–3000, a monthly salary above of EUR 3000), study programme (science and technology, humanities, social sciences, and art), and educational stage (baccalaureate and other). For the three sections remaining of the final questionnaire, which consisted of 22 items, we used a Likert scale with values ranging from 1 to 4 (1, “not at all”; 2, “somewhat”; 3, “quite a bit”; and 4, “very much”) to avoid middle-ground answers.

### 2.5. Data Analysis

In order to ensure data content validity, we performed content analysis, obtaining the consensus of a group of experts using the Delphi technique [47]. Construct validity was tested by means of descriptive statistics and internal consistency estimates through exploratory analysis, multivariate analysis, and decision tree using SPSS 24.0 (IBM SPSS Statistics 24.0, Chicago, IL, USA, 2016) [55] and confirmatory analysis using LISREL v9.1 (Scientific Software International, Princeton, NJ, USA, 2010) [56].

## 3. Results

In Table 2, all items show positive asymmetry, which meant that the students tended to use the low end of the scale, except for the following items: mother’s employment status and influence of the student’s interests. These two items showed negative asymmetry, which meant that the students tended to use the high end of the scale.

Regarding kurtosis analysis, more than half of the indices were negative: mother’s employment status; father’s educational level; mother’s educational level; programme chosen in the fourth year of compulsory secondary education (CSE); chosen Baccalaureate programme; parental influence on university degree choice; influence of friends; marks and results obtained in the PEvAU; “I am able to make my own decisions on the basis of my goals”; “I am confident in defining short- and long-term future goals, and organising myself to achieve them”; “when I have a problem, I try to solve it and learn from that experience”; “I am motivated to advance my goals, without anyone imposing them on me”; and “I consider myself capable of developing new projects for the future; my decision-making to achieve my goals depends on the goals of my friends”. This indicates that the points were less clustered and below the normal distribution curve. The remaining items were positive: father’s marital status; father’s employment status; influence of optional subjects chosen in fourth year of CSE; decision to take the PEvAU; preferred university degree; influence of student’s own interests and the selection of the baccalaureate programme according to university degree choice; “my higher studies will make my family proud of me”; “career realisation is to achieve greater socioeconomic projection”; and “I study higher studies for family taxation”, which indicated that the points were more clustered and lay above the curve of normal distribution.

Table 3 shows multivariate ANOVA (MANOVA) results in relation to the variables of gender and socioeconomic status, and their interaction. Effect size was calculated using η^2^. 

A multilevel test was used to simultaneously analyse the relationship between different levels of the same variable and other variables with various levels [57], which enabled us to statistically assess the influence of gender variables (with two levels) and socioeconomic status (with three levels) on the individual level. This allowed for us to study the covariance effect of these variables and their inter-relation. 

Results indicated significant differences and large effect sizes with respect to career guidance, which demonstrated its importance in decision-making processes concerning higher education and career choice. The sample size or the proportion of explained variance (MANOVA) [58,59] indicated that, with respect to the family influence factor, more than 26% (η^2^ = 0.261) of the found differences could be attributed to the effects of the interaction between gender and socioeconomic variables. Likewise, the obtained results showed significant differences with respect to the factor of “how does it influence decision-making?”, and sample size (MANOVA) [58,59] indicated that 37% (η^2^ = 0.37) of the found differences could be attributed to the effect of the interaction between levels, of which the value was one-third of the surveyed population. In social terms and according to the study, this is a value to consider. Regarding the square sample size in relation to the factor “reasons for pursuing higher education”, 21% (η^2^ = 21) of the found differences were attributed to the effect of the difference in relation to gender and socioeconomic status. 

**Figure 1:**
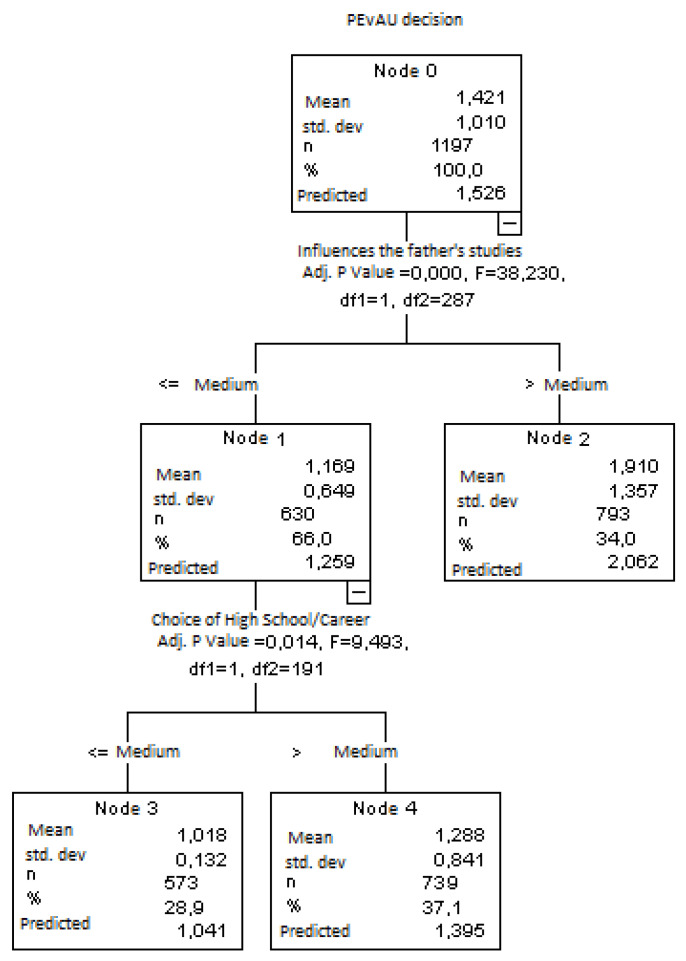
Classification tree for university entrance examination (PEvAU).

As shown in Figure 1, we graphically and analytically researched on the basis of a decision tree, which helps to understand behaviour with respect to a certain decision and reduces the number of independent variables [60].

In this case, we wanted to find the most influential factors when deciding whether to take the PEvAU. That is, we intended to analyse the characteristics that could influence secondary education and, later, Spanish Baccalaureate students in their decision to take this exam. 

Figure 1 indicates that the variable “parental influence on university degree choice” is the best predictor of the decision to take the PEvAU. From here, it splits into Nodes 1 and 2. Node 1 shows that 60.6% of the students were influenced by their parents in their university degree choice compared to the results of Node 2, where 39.4% were not.

Node 1 split again into Nodes 3 and 4, associated with the variable “relationship between baccalaureate programme and students’ university degree choice”. In Node 3, 41.3% admitted that they were related, compared to 19.2% in Node 4 who did not see any relationship between the baccalaureate programme of choice and the university degree that they wished to study for.

## 4. Discussion

Our main results indicated a significant influence of parents on the choices of their children, with the decision to take the PEvAU being the best predictor. These were students who were transitioning from the Spanish Baccalaureate to university, and they depend on the career guidance that they received in secondary education in relation to their academic choices since the difficulty of the decision is exacerbated by internal barriers, such as lack of motivation, and external barriers, such as a lack of information and disorientation [24,40,61,62]. 

The adjustment of results from MANOVA data revealed that future career and higher-education choices based on gender and socioeconomic status are significant with respect to family influence (*p* < 0.000), “how does it influence decision-making?” (*p* < 0.000), and “reasons for pursuing higher education” (*p* < 0.000). Therefore, both gender and socioeconomic status are key factors determining both the university degree that students choose and the decision of whether to continue their studies in higher education.

However, given the results of this study, university degree choices depend on the Spanish Baccalaureate programme followed by the students. Parents do not have a direct influence on their children regarding what exactly to study at university, but they do have an influence on the enhancement of self-confidence and self-efficacy in the process [10,11,19], strengthening self-esteem, and the importance of “me” through success [63], as evidenced by our conclusion that the decision to take the PEvAU is influenced by families, and specifically parents. They influence career decision-making regardless of whether each individual goes through processes of both formal and informal learning, as well as self-directed learning, in the sense that they structure their own decision-making as they progress through their own journey of figuring out how to best make their own decision [64].

Nevertheless, the influence of parents on children presents some differences according to gender. Thus, mothers have more influence on their daughters [39], especially when compared to the influence that fathers have on their sons. Although sons are more influenced by their fathers, and daughters by their mothers, this rule does not deny the existence of studies where this relationship between parents and children is not confirmed [65]. This information has not been fully confirmed by Spanish research studies. The closest example is the study by Cortes and Conchado [24] that found that men generally feel less influenced by parents (70.0%) than women (78.6%) do. However, it did not provide information about the gender of parents in order to understand if this difference was found in both cases, or in the case of mothers or fathers. Research by Rodríguez, Peña, and Inda [30] concluded that, when one parent is regarded as the leading figure, in most cases it is the mother. 

It was also verified that the selection of the baccalaureate programme is significantly related to the university degree of choice. Of the students, 80.8% justified their said choice on the basis of their career interests or preferences. That is, their baccalaureate option was closely related to what they chose to study; therefore, they decided to take the PEvAU. 

However, decision-making is strongly driven by the educational level of parents as a factor that influences students. Of mothers, 24% hold the Spanish Baccalaureate diploma, and 25.7% have had higher education; among fathers, 26.2% hold the Spanish Baccalaureate diploma, and 30.7% have had higher education. Considering this, the educational decision to continue studies in higher education is both linked to having successfully completed CSE, the Spanish Baccalaureate, and the PEvAU, and to their preference of attaining a high level of education, similar to that of their parents, in order to avoid the risk of downward social mobility [65]. 

The need to analyse the academic decision-making process is one link in the chain of career decisions that students make throughout their life course [66].

Since psychosocial factors that influence a student’s decision-making that is developed in the second factor obtained results that showed that, even though there were no differences between genders, there were differences in relation to socioeconomic level, with the low profile being 26%, the one who is guided more by their peer group and has less motivation—which influences their progress in life, perception of difficulty, and performance [67]; the majority level, with an average socioeconomic level of 58%—develops better values of self-concept, motivation, and self-esteem, and greater influence of family type [68].

Regarding the influence of other social agents in the academic decision-making process, 36.3% of students reported to be influenced (somewhat, very much, quite a bit) by their friends, that is, they recognised that friends are an important source of support, but they do not have enough influence to change their decisions. This result is consistent with the findings of previous research [35,38,69].

As a future line of research and an implication of our results, there is a need to continue researching career guidance from secondary education onwards given the limited available information. It would be interesting to analyse, depending on the cultural environment of the students, factors that can influence student decision-making and explore how the career decision-making process is performed according to the new family format.

## 5. Conclusions

Our society believes that university studies should be pursued after completing the Spanish Baccalaureate because it will be needed tomorrow. We grow up thinking this, and it socialises us into this viewpoint. However, when the time comes to make an academic decision, we need to know whether this choice is the right one.

Among influences perceived by students, parents stand out as the most dominant, being acknowledged by a large majority of students [24]. However, the influence of parents on children presents differences according to gender. Thus, mothers have more influence on daughters [29,39], especially when compared to the influence that fathers have on their sons, although sons are more influenced by their father, and daughters by their mothers. This rule does not deny the existence of studies where this relationship between parents and children is not confirmed [64]. From psychosocial factors that influence student decision-making, adolescents are indecisive, which in some cases makes parents lead their children to choosing a career that does not fit their personality [9,10,11,12]. In contrast, our study concluded that medium–high socioeconomic level positively affects their self-concept, motivation, and self-esteem. Although there are no differences between genders, there are in relation to socioeconomic level.

When asked about their choice, those involved recognised the influence of their parents and described it as a determining factor that is not comparable in importance to the other possibilities (friends, teachers, siblings, tutors, etc.) [24].

## Figures and Tables

**Table 1:** Model adjustment index of Educational Career Guidance Tool for Pre-University Student Questionnaire (EGPUQ).

			Absolute Adjustment Index			Increased Adjustment Index			
Model	CMIN	P	LO 90	HI 90	RMSEA	PNFI	NFI	CFI	TLI
3 factor	834.6	0.00	0.044	0.076	0.045	0.730	0.935	0.928	0.926

Note: RMSEA, root mean square error of approximation; CFI, comparative fit index; CMIN, related chi-squared statistics, PNFI, parsimony normed fit index, NFI, normed fit index, TLI, Tucker–Lewis index.

**Table 2:** Asymmetry, kurtosis, and Kolmogorov–Smirnov Z for EGPUQ. Note: PEvAU, university entrance examination.

	Asymmetry	Kurtosis	Z (*)	*p* (**)
Father’s marital status	2.028	2.778	6.836	0.000
Father’s employment status	1.853	1.447	7.144	0.000
Mother’s employment status	–0.020	–2.019	4.927	0.000
Father’s educational level	0.328	–0.852	2.593	0.000
Mother’s educational level	0.444	–0.863	2.799	0.000
Programme chosen in fourth year of compulsory secondary education	0.423	–1.716	5.217	0.000
Influence of optional subjects chosen in fourth year of compulsory secondary education	2.630	1.252	6.865	0.000
Baccalaureate programme chosen	0.474	–0.925	5.095	0.000
Decision to take PEvAU	1.963	1.977	7.251	0.000
Parental influence on university degree choice	0.001	–1.021	2.107	0.000
Influence of friends	0.623	–0.441	3.093	0.000
Influence of students’ own interests	–1.457	1.512	4.287	0.000
Student’s marks	0.040	–1.014	4.938	0.000
PEvAU results not high enough to gain admission to their chosen degree	0.751	–0.850	3.871	0.000
Relationship between baccalaureate programme and university degree choice	1.211	0.389	5.744	0.000
“I am able to make my own decisions on the basis of my goals”	0.381	–1.671	5.690	0.000
“I am confident in defining short- and long-term future goals, and organising myself to achieve them”	0.351	–1.168	6.430	0.000
“When I have a problem, I try to solve it and learn from that experience”	0.186	–1.066	2.358	0.000
“I am motivated to advance my goals without anyone imposing them on me”	0.140	–1.002	3.123	0.000
“I consider myself capable of developing new projects for the future; my decision-making to achieve my goals depends on the goals of my friends”	0.616	–0.910	2.451	0.000
“My higher studies will make my family proud of me”	1.011	0.654	3.984	0.000
“Career realisation is to achieve greater socioeconomic projection”	0.887	1.021	4.971	0.000
“I study higher studies for family taxation”	0.447	1.101	4.532	0.000

Note: * Z= Kolmogorov–Smirnov Z; ** *p* = statistical significance (bilateral).

**Table 3:** Multivariate ANOVA (MANOVA) and effect size (η^2^) sums of aggregated scales for EGPUQ by gender and socioeconomic status.

Factors		M	SD	CI (95%)		*F*	*p*	*η* ^2^
				Lower Limit	Higher Limit			
Family influence	Gender	4.18	0.812	3.89	4.34	0.887	<0.000	0.32
	Socioeconomic status	4.12	0.686	3.43	4.31	1.721	<0.000	0.21
	Gender × Socioeconomic status	4.04	0.987	3.67	4.12	1.122	<0.000	0.26
How does it influence decision-making?	Gender	4.03	0.918	3.52	4.29	1.127	>0.000	0.06
	Socioeconomic status	4.01	0.996	3.43	4.11	1.115	<0.000	0.07
	Gender × Socioeconomic status	4.12	0.983	3.34	4.35	1.006	<0.000	0.37
Reasons for pursuing higher education	Gender	3.65	1.010	3.21	3.83	0.818	>0.000	0.42
	Socioeconomic status	3.82	1.086	3.32	3.98	1.846	<0.000	0.39
	Gender × Socioeconomic status	3.61	1236	3.28	4.05	1.993	<0.000	0.21

Note: Critical alpha level was adjusted for multiple testing to reduce type I error (α), and thus α value was divided by number of pair comparisons for each ANOVA.

## Data Availability

The data presented in this study are available on request from the corresponding author.
